# Discovery of piRNAs Pathway Associated with Early-Stage Spermatogenesis in Chicken

**DOI:** 10.1371/journal.pone.0151780

**Published:** 2016-04-05

**Authors:** Lu Xu, Lingling Qiu, Guobin Chang, Qixin Guo, Xiangping Liu, Yulin Bi, Yu Zhang, Hongzhi Wang, Zhiteng Li, Xiaoming Guo, Fang Wan, Yang Zhang, Qi Xu, Guohong Chen

**Affiliations:** 1 College of Animal Science & Technology, Yangzhou University, Yangzhou, Jiangsu, 225009, China; 2 Poultry Institute, Chinese Academy of Agricultural Sciences, Yangzhou, Jiangsu, 225003, China; Qingdao Agricultural University, CHINA

## Abstract

Piwi-interacting RNAs (piRNAs) play a key role in spermatogenesis. Here, we describe the piRNAs profiling of primordial germ cells (PGCs), spermatogonial stem cells (SSCs), and the spermatogonium (Sp) during early-stage spermatogenesis in chicken. We obtained 31,361,989 reads from PGCs, 31,757,666 reads from SSCs, and 46,448,327 reads from Sp cells. The length distribution of piRNAs in the three samples showed peaks at 33 nt. The resulting genes were subsequently annotated against the Gene Ontology (GO) database. Five genes (*RPL7A*, *HSPA8*, *Pum1*, *CPXM2*, and *PRKCA*) were found to be involved in cellular processes. Interactive pathway analysis (IPA) further revealed three important pathways in early-stage spermatogenesis including the FGF, Wnt, and EGF receptor signaling pathways. The gene *Pum1* was found to promote germline stem cell proliferation, but it also plays a role in spermatogenesis. In conclusion, we revealed characteristics of piRNAs during early spermatogonial development in chicken and provided the basis for future research.

## Introduction

Great losses have been reported for farms due to the large number of chickens that suffer from azoospermia; however, it has so far been difficult to elucidate the mechanism of spermatogenesis in chicken. Recently, researchers have suggested that Piwi-interacting RNAs (piRNAs) play a key role in this process. PiRNAs are non-coding RNAs of 24–35 nucleotides (nt) in length that were first discovered in 2006 and are enriched in animal gonads, where they repress transposons to maintain genome integrity [1,2]. In the porcine testis, the Piwi/piRNA-mediated post-transcriptional silencing pathway plays a conserved role in mammalian spermatogenesis [3,4]. In mice, Lim et al. defined a critical role for HEN methyltransferase 1 and piRNAs in the maintenance of transposable element repression in adult germ cells and in setting the spermatogenic program [5]. In developing *Drosophila* ovaries, secondary piRNA-guided target slicing is the predominant mechanism that specifies transcripts, including those from piRNAs clusters, as primary piRNAs precursors and defines the spectrum of Piwi-bound piRNAs in germline cells during oogenesis. Additionally, target slicing defines the nuclear piRNAs pool during spermatogenesis in mice [6]. Moreover, during early meiosis, the transcription factor *A-MYB* initiates pachytene piRNAs production and regulation of piRNAs pathway proteins and piRNAs genes create a coherent feed-forward loop that ensures the robust accumulation of pachytene piRNAs in mouse testes [7]. In mice, pachytene piRNAs are the end-products of RNA processing during spermiogenesis by RNA-seq [8]. During meiosis, piRNAs populations are selected to enable successful spermatogenesis, both driving the response away from essential genes and directing the pathway toward mRNA targets that are regulated by small RNAs in meiotic cells [9]. Furthermore, in the mouse elongating spermatid phase, pachytene piRNAs are responsible for massive mRNA elimination and inactivating vast cellular programs in preparation for sperm production [10]. Therefore, a large number of reports have suggested that piRNAs are involved in spermatogenesis, but few reports can be found in poultry. We used chicken as a model to find the relationship between piRNAs and spermatogenesis. Here, we analyzed the piRNAs profile in three types of cells–PGCs), SSCs, and Sp during the early spermatogenesis stage. We aimed to find several key piRNAs that are involved in germ cell and spermatogonial development via small RNA sequencing and to understand these processes from an epigenetic perspective.

## Materials and Methods

### Samples

All animal experimental procedures were approved and guided by the Institutional Animal Care and Use Committee of the School of Animal Science and Technology, Yangzhou University (Permit Number: 45, Government of Jiangsu Province, China) and the U.S. National Institute of Health guidelines (NIH Pub. No. 85–23, revised 1996).

We purchased 25 male Langshan chickens at 28 weeks of age and 2000 freshly fertilized eggs from the Poultry Institute, Chinese Academy of Agricultural Sciences (Yangzhou, China). Half of the eggs were used to isolate PGCs from the gonads of chicks hatched for 5.5 days (Stage 28) and SSCs from the left testis of chicks hatched for 18 days. PGCs and SSCs were separated by density gradient equilibrium centrifugation and the differential adhesion method [11,12]. PGC colonies were identified using the mouse anti-chicken c-Kit antibody stain (Santa Cruz Biotechnology, Santa Cruz, CA, USA; 1:50); the second antibody was a goat-anti-mouse IgM-FITC antibody (Santa Cruz Biotechnology, Santa Cruz, CA, USA; 1:50). SSCs were identified using a rabbit polyclonal antibody to Integrin alpha 6 conjugated to FITC (Biorbyt Biotechnology, Biorbyt, United Kindom; 1:50) according to each manual provided with each reagent. Sp were isolated from the testis of cocks and were sorted by flow cytometry [13].

PGCs and SSCs were screened with the c-Kit antibody and Integrin alpha 6 antibody, respectively, using a FACSAria flow cytometer (FACSAria, BD Biosciences, San Jose, CA, USA). According to a method by Chang et al., the Sp single-cell suspensions from testicular tissues were prepared and isolated by flow cytometer [14]. Sp were dyed with 2-(4-amidinophenyl)-6-indolecarbamidine dihydrochloride (DAPI) (Beyotime Biotechnology, Shanghai, China) during flow cytometry.

According to the viewpoint of animal welfare, 25 male Langshan chickens were stay in 25°C (average temperature) environment. Each chicken feed in one cage and volume of cage was 35*38*42 centimeters. They feed with complete diet three times a day. The treatment of 25 chickens were as followed: firstly, we use Xylazine Hydrochloride Injection to anesthetize the chicken with the volume of medical was 0.4ml/kg. After 3 minutes, two wings and tail feathers were fall down. 8 minutes later, the eyes were closed and fall into the sleep. Secondly, cut off the right abdomen feathers with surgical scissors and clean the skin with alcohol. Then used scalpel to gash skin tissues and find the testis. Finally, we performed wound closure to chicken. It would take 30 minutes to finish this surgery and the chicken kept sleeping during surgery. Thirdly we used brachial vein injection to treat with chicken euthanasia. Find brachial vein under the wing and clean the skin with alcohol to make the vein expand, then we inject air into the vein with injector. According to Animal Management Rules of Yangzhou University, we transfer these chickens to that office. All the treatment were performed in aseptic environment and we covered with protection suit.

### Small RNA library construction and sequencing

According to the mirVana^™^ miRNA Isolation Kit (Ambion, Austin, TX, USA) manual, we isolated all samples of total RNA. Approximately 3 μg total RNA from each test sample (PGCs, SSCs, and Sp) was used for small RNA sequencing. The quality of RNA samples was measured by an Agilent 2100 Bioanalyzer (Agilent technologies, Santa Clara, CA, USA). Small RNA fractions were ligated to 5′ and 3′ adaptors. After amplification, the three cell libraries (140–150 bp) underwent quantification and quality assessment by Qubit^®^ 2.0 Fluorometer and Agilent 2100 Bioanalyzer (Agilent technologies, Santa Clara, CA, USA). Small RNA sequencing was performed on the HiSeq 2500 platform at Shanghai Biotechnology Co., Ltd (Illumina, Shanghai Biotechnology Co., Ltd, China). The initial sequencing results were converted into sequence data by base calling to generate raw data.

### Bioinformatics analysis

Raw data were processed to obtain clean reads by filtering adaptor-ligated contaminants, low-quality reads (Q-value < 20), and short read tags (< 18 nt) using Fastx (fastx_toolkit-0.0.13.2, FastQC: http://www.bioinformatics.babraham.ac.uk/projects/fastqc/). Briefly, to identify miRNAs, the reads were aligned with the miRbase, ncRNA, and Rfam databases using CLC genomics workbench. We then set three capture premises to investigate the piRNAs by Excel: (a) inclusion of the phrase “-binding small RNA”, (b) length of 26–39 nt, and (c) count ≥ 30. We used DEGseq R and perl to analyze differentially expressed piRNAs. We analyzed these data from two sides. First, we performed a Venn diagram analysis of common piRNAs in the three types of cells. We verified these piRNAs by piRNAspredictor (http://122.228.158.106/piRNAs/analysis.php) after searching in the database [4]. We mapped these sequences onto the reference genome from the NCBI (*Gallus gallus* v.4) using Bowtie 2.0. Gene expression levels were calculated by the TPM (transcripts per million) method. Gene Ontology (GO) and Kyoto Encyclopedia of Genes and Genomes (KEGG) pathway analyses were performed on these genes. Gene Ontology (GO) analyses of differentially expressed genes (DEGs) were based on DAVID (https://david.ncifcrf.gov/) and WEGO (http://wego.genomics.org.cn/cgi-bin/wego/index.pl). KEGG pathways were based on KOBAS 2.0 (http://www.kobas.cbi.pku.edu.cn/home.do) and SBC Analysis System (SAS, Shanghai Biotechnology Co., Ltd, Shanghai, China). An interactive pathway analysis (IPA) was performed using the Ingenuity software (http://www.ingenuity.com). Secondly, we defined two groups (PGCs vs. SSCs and SSCs vs. Sp). We performed a Venn diagram analysis to identify common unique piRNAs in the two groups. We verified these piRNAs with piRNAspredictor after searching in the database. We mapped these sequences onto the reference genome from NCBI (*Gallus gallus* v.4) using Bowtie 2.0. GO and KEGG pathway analyses were also performed on these genes, as above.

### Quantitative RT-PCR

Quantitative RT-PCR (RT-qPCR) using SYBR green (Takara) was performed according to the manual. RT-qPCR was performed on three samples for each candidate gene. GAPDH, a housekeeping gene, was used as a control. Reverse transcriptase reactions included 1 μg of total RNA per sample. Quantitative PCR reactions were used to calculate the relative fold-change in accordance with the ΔΔCT method. Where appropriate, comparisons of gene expression levels were analyzed by ANOVA using SPSS19.0 and visualized with SigmaPlot 12.5.

## Results

### Identification of cells

**Fig 1:**
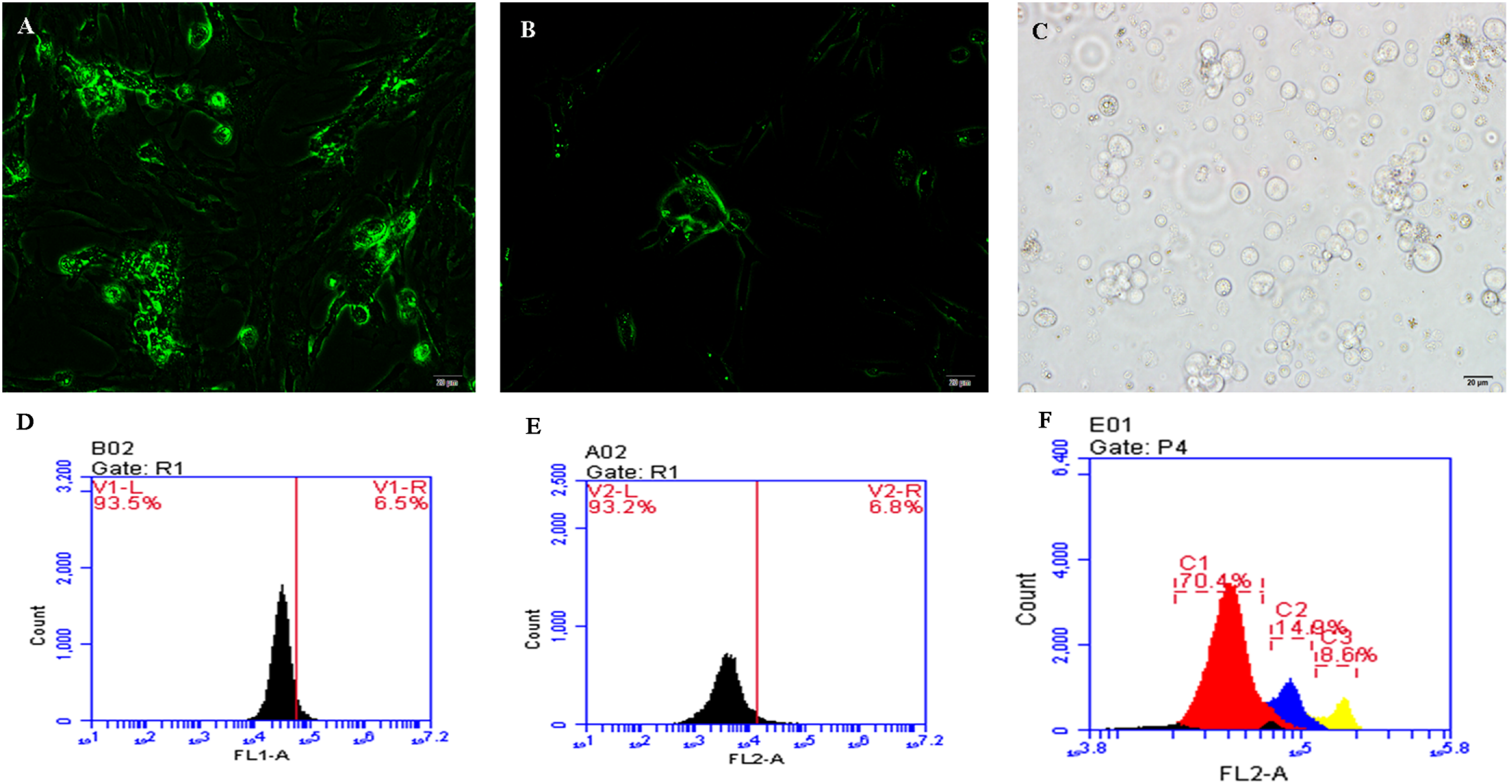
Cell sorting via flow cytometry. (A) The primordial germ cells (PGCs) were identified with a c-Kit antibody marked with FITC (200×). (B) The spermatogonial stem cells (SSCs) were marked with an Integrin alpha 6 antibody with FITC (200×). Both PGCs and SSCs were cultured for 48 h. (C) The spermatogonial (Sp) cells were directly isolated from testes and identified under light microscopy (200×). Scale bar = 20 μm. (D-F) Flow cytometry sorting for PGCs (D), SSCs (E), and Sp cells (F). The PGC, SSC, and Sp sorting ratios were 6.5%, 6.8%, and 14.9% respectively. Sp cells were stained with DAPI.

We used flow cytometry to identify the three types of cells (Fig 1). The PGCs were large cells and became a mulberry-like shape after being cultured for 48 h, while the SSCs became bird nest-like after 48 h. The Sp cells were isolated from the testes of sexually mature cocks and were larger than the other two types of cells. Three peaks can be seen in Fig 1F, reflecting haploid, diploid, and tetraploid cells. We collected the diploid cells (second peak) for sequencing.

### Sequencing results

**Fig 2:**
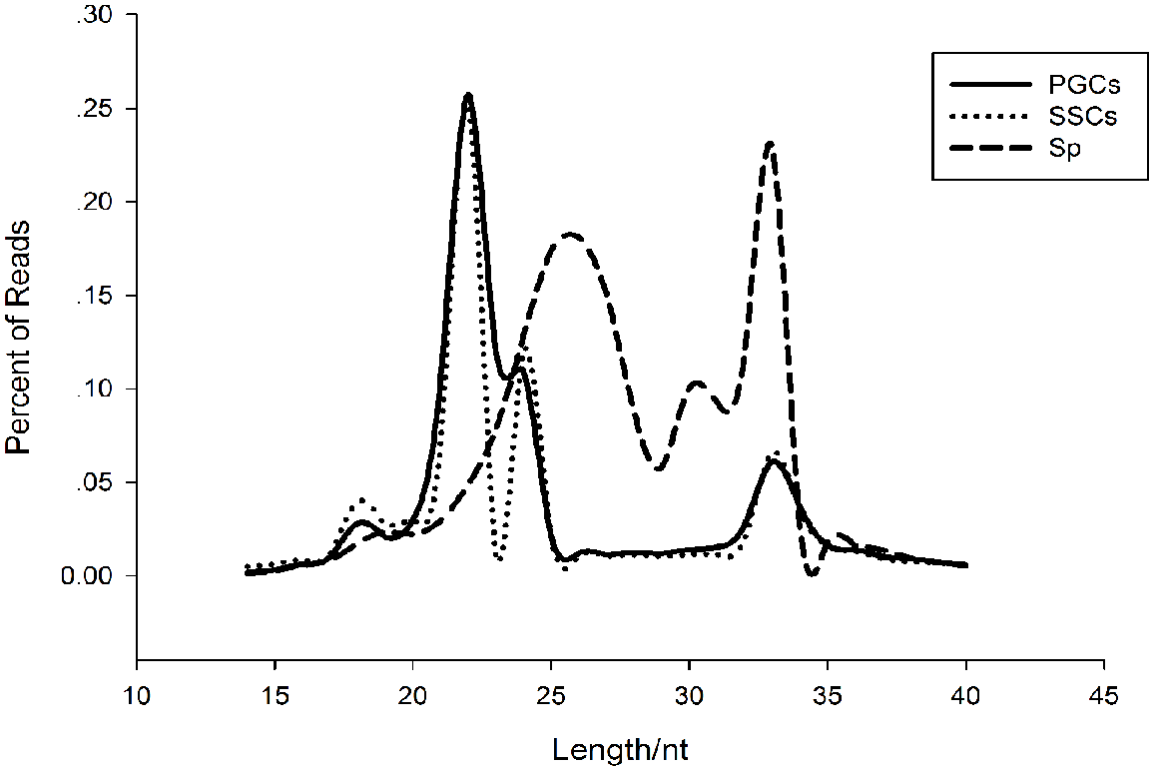
Length distribution of small RNAs in the three types of cells.

**Fig 3:**
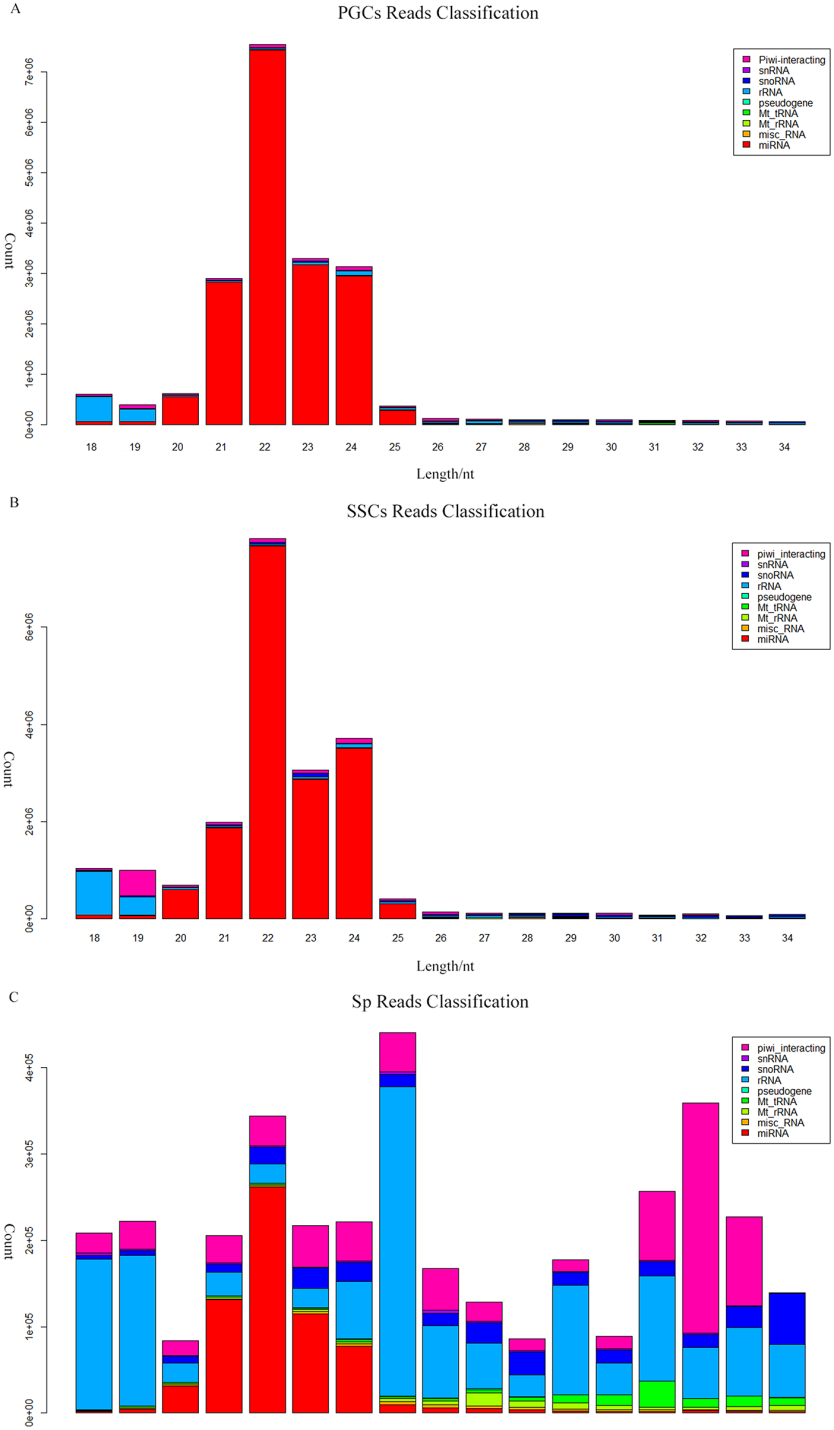
Classification and annotation of small RNAs from different cell types. Small RNAs from (A) PGCs, (B) SSCs, and (C) Sp cells were shown. Different small RNA groups were represented by different colors.

We obtained 31,361,989 reads from PGCs, 31,757,666 reads from SSCs, and 46,448,327 reads from Sp cells, representing an effective ratio of > 95% (Table 1). Examining the length distribution of the reads, we found that all the samples have two peaks, both PGCs and SSCs showed one peak at 22 nt and one at 33nt, while the Sp distribution showed one peak at 26 nt and one at 33 nt (Fig 2). According to Chen et al. and Juliano et al., the 22-nt peak represents mainly microRNAs, whereas from 26-nt to 33-nt peaks represent piRNA clusters [15, 16]. We were able to classify and annotate 9 groups of small RNAs (Fig 3), with different distributions appearing in different cell types. The 9 groups represent miRNAs, misc_RNAs, mt_rRNAs, mt_tRNAs, pseudogenes, rRNAs, snoRNAs, snRNAs, and piRNAs. The number small RNAs found in each group is shown in S1 Table.

**Table 1:** Number of reads obtained for the three sample libraries.

Sample	Clean Reads	Effective reads	Effective ratio
PGCs	31361989	30851348	98.37%
SSCs	31757666	31461002	99.07%
Sp	46448327	46273957	99.62%

### Common piRNAs in the three cell types

#### Venn diagram analysis

**Fig 4:**
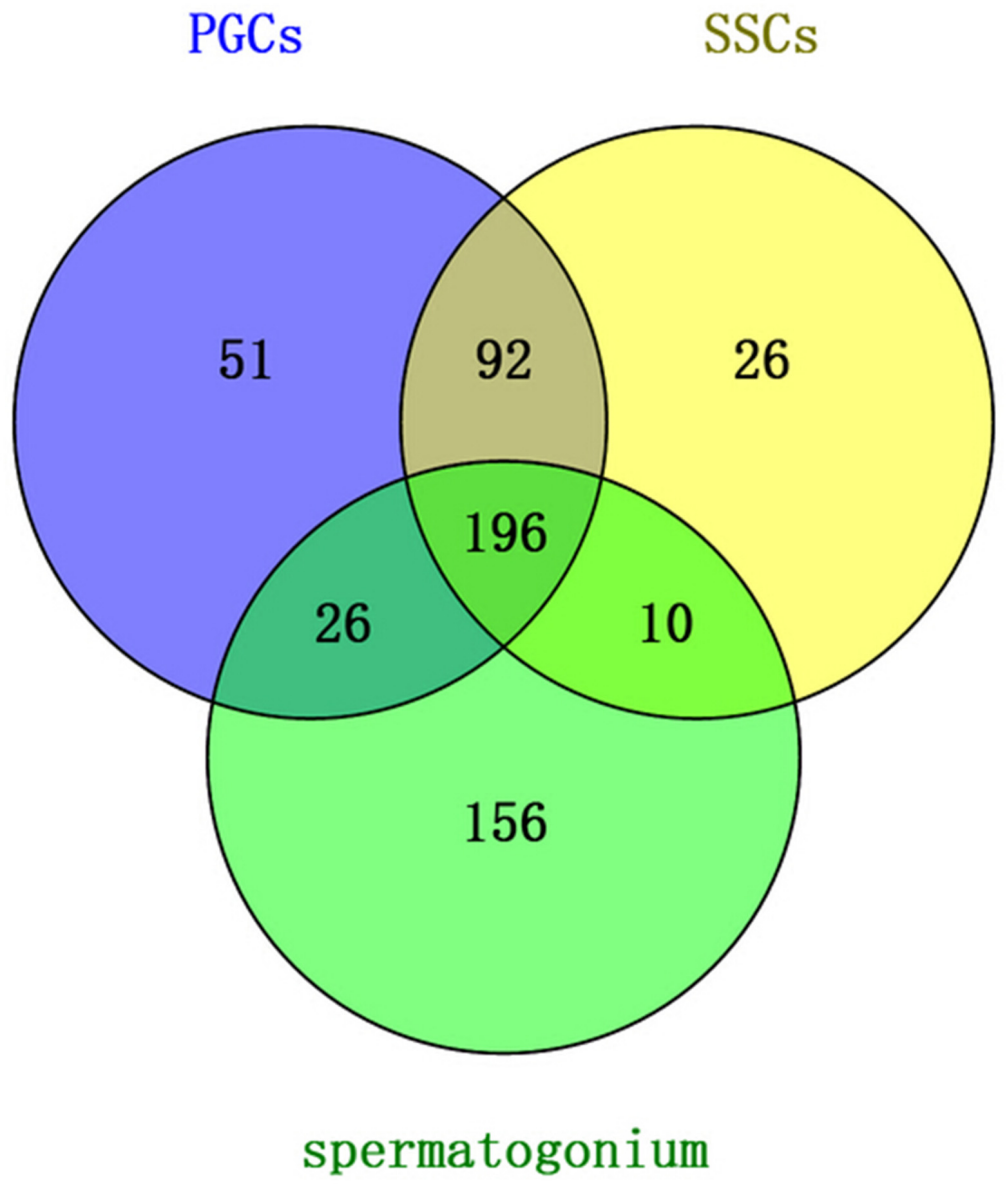
Venn diagram comparing piRNAs among the three cell types.

Venn diagram analysis revealed that 196 unique piRNAs appeared in all three cell types. Of these, the PGCs contained 365 total reads, the SSCs contained 324 reads, and the Sp cells contained 388 reads. Only 10 piRNAs were present in SSCs and Sp but not PGCs, while 92 reads were present in PGCs and SSCs only and 26 reads were present in PGCs and Sp only (Fig 4). The uploading of the 196 common piRNAs to piRNAspredictor software showed that 153 of these piRNAs could be verified (S2 Table).

#### GO and KEGG pathway analyses

**Fig 5:**
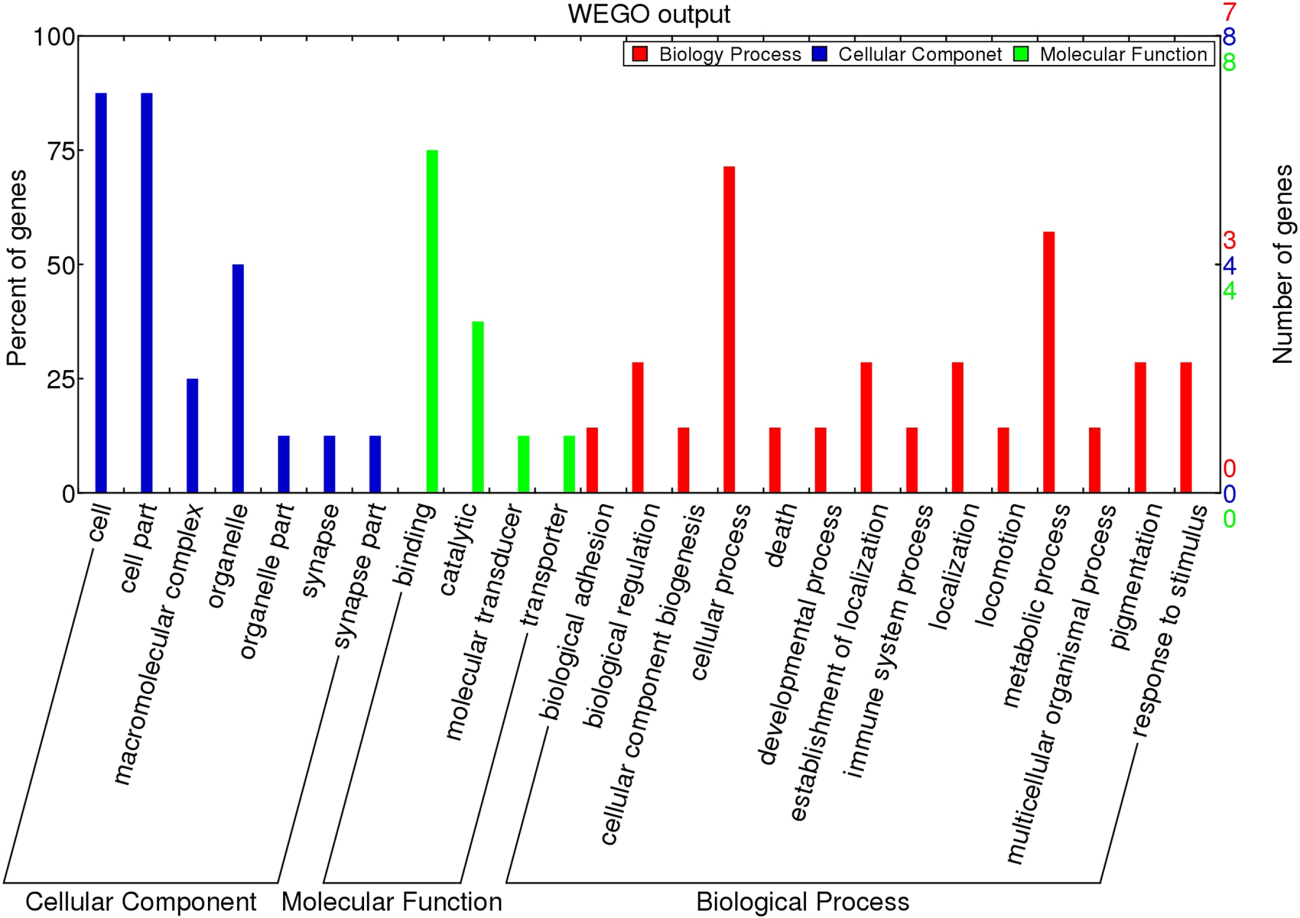
Gene Ontology classification of piRNAs target genes which were found in all three cell types (PGCs, SSCs, and Sp cells).

**Fig 6:**
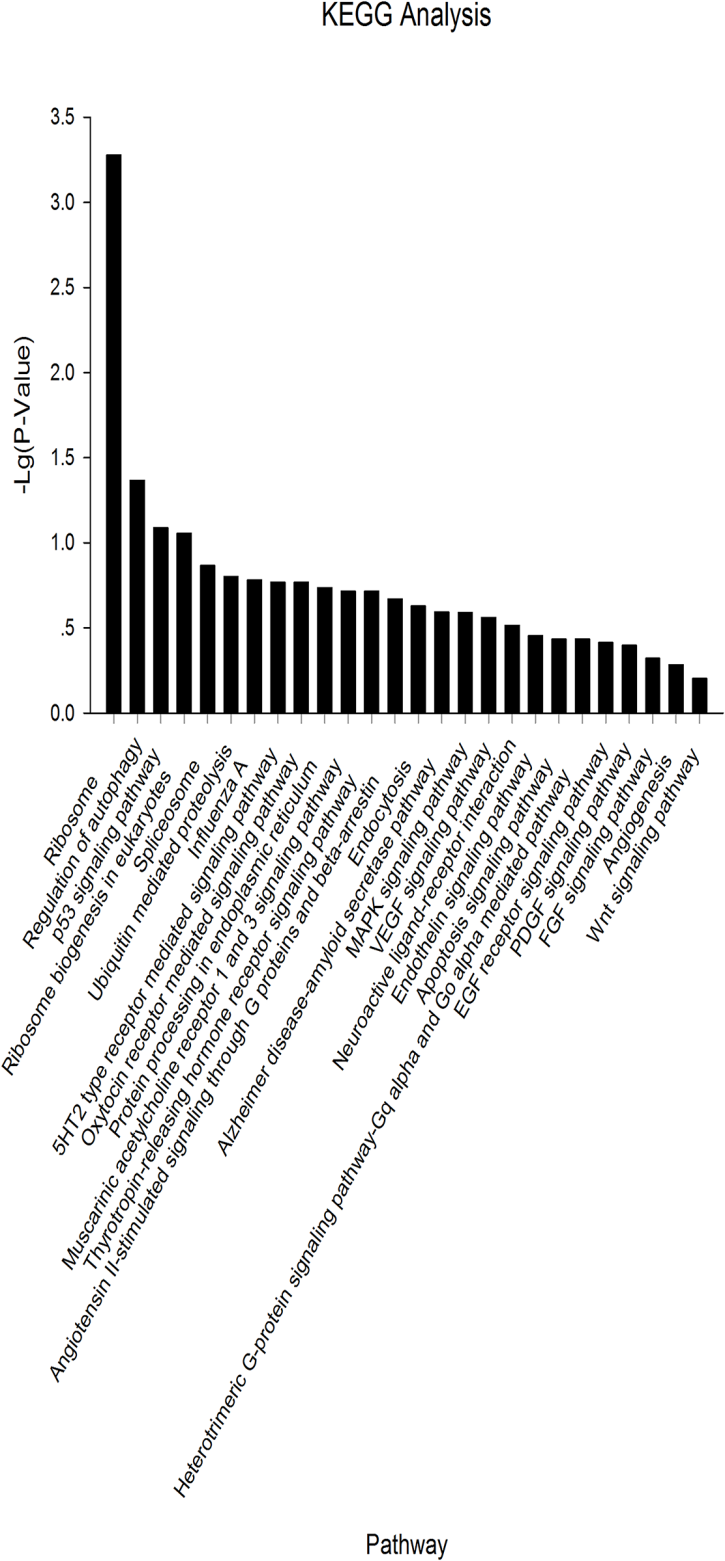
KEGG analysis of piRNAs common to all three cell types (PGCs, SSCs, and Sp cells).

We mapped the 153 validated piRNAs sequences onto the chicken reference genome (*Gallus gallus* v.4) using Bowtie 2.0. After filtering for repeats, 22 genes were annotated (S3 Table). Gene Ontology (GO) analysis of these genes showed that nearly 75% of the genes are related to cellular processes (including *RPL7A*, *PRKCA*, *PUM1*, *HSPA8*, and *CPXM2*) while 20% of the genes are involved in developmental processes within the Biological Process category. Within the Cellular Component category, nearly 80% of the genes contained the ontology “cell part”. For the Molecular Function category, 75% of the genes were associated with binding, including ATP binding, nucleotide binding, or metal ion binding (Fig 5). In the KEGG pathway analysis, 26 pathways were identified. Of these, 9 pathways were found to be involved in cellular processes, including migration, proliferation, development, and signal transduction (Fig 6).

### Different expressed piRNAs in two groups

#### Venn diagram analysis

**Fig 7:**
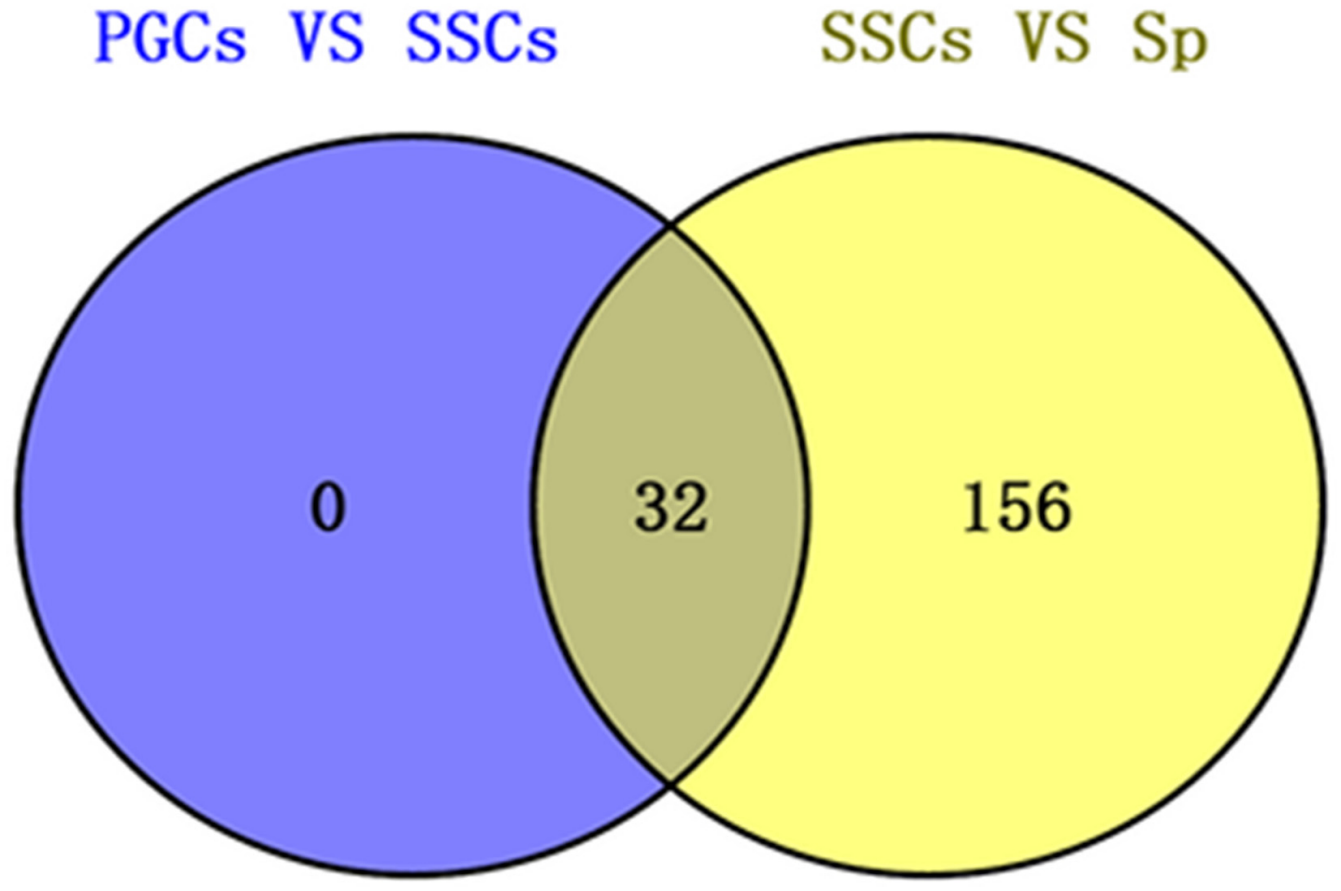
Venn diagram analysis of common different piRNAs in two groups (PGCs vs. SSCs and SSCs vs. Sp).

**Fig 8:**
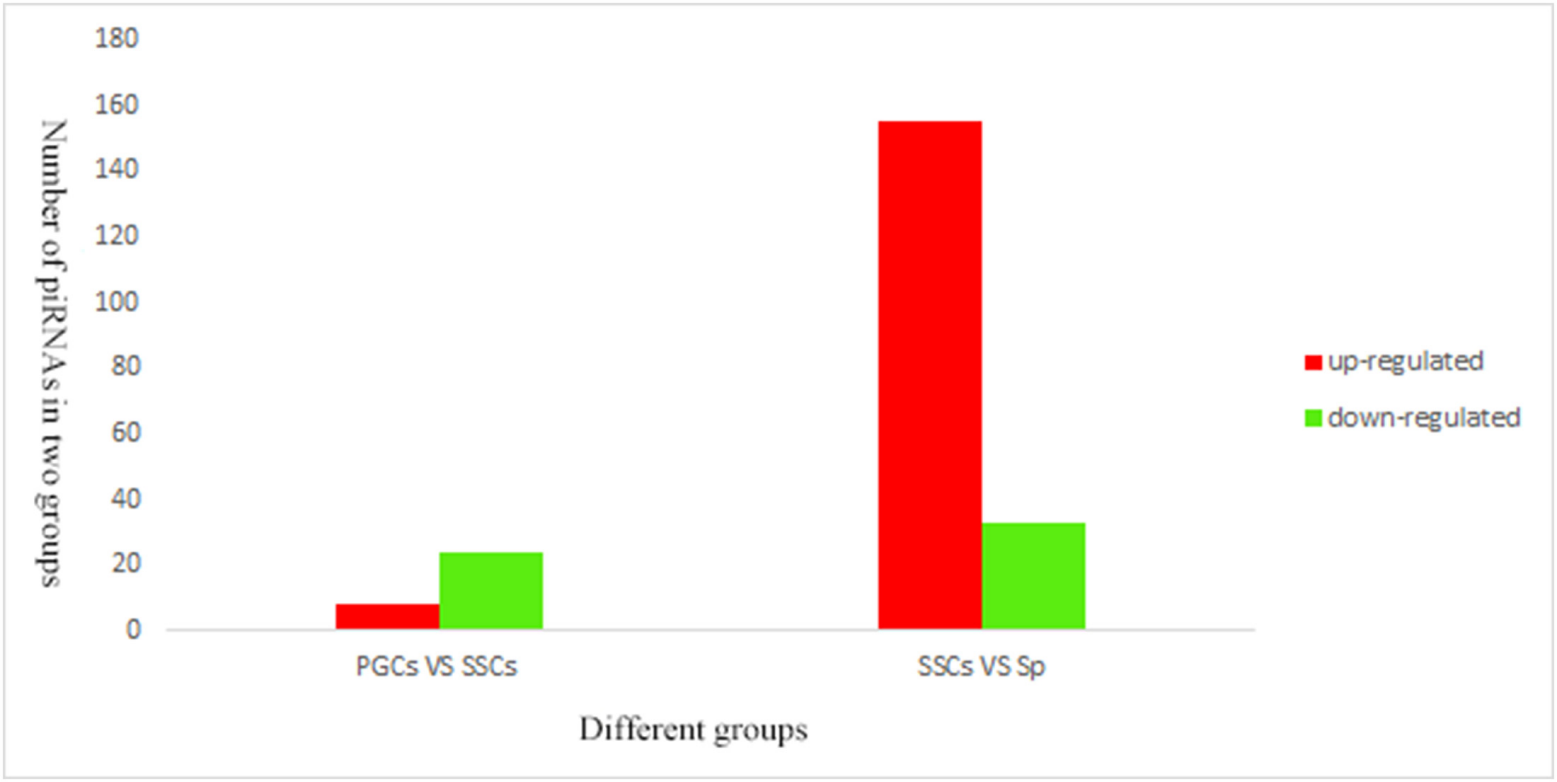
Number of piRNAs up- or down-regulated among two groups (PGCs vs. SSCs and SSCs vs. Sp). The x-axis represents the number of piRNAs, and the y-axis reflects the groups.

There were 32 common different expressed piRNAs that were identified in two groups (PGCs vs. SSCs and SSCs vs. Sp). The PGCs vs. SSCs group contained no unique common different piRNAs, while the SSCs vs. Sp group contained 156 common different piRNAs (Fig 7). Of these, 32 piRNAs overlapped with the former group of 153 validated piRNAs that were common to all three groups (S4 Table). The number of differnet expressed piRNAs in two groups showed in Fig 8. In the PGCs vs. SSCs group, 8 piRNAs were up-regulated and 24 were down-regulated. In the SSCs vs. Sp group, 155 piRNAs were up-regulated and 33 were down-regulated.

#### GO and KEGG pathway analyses

**Fig 9:**
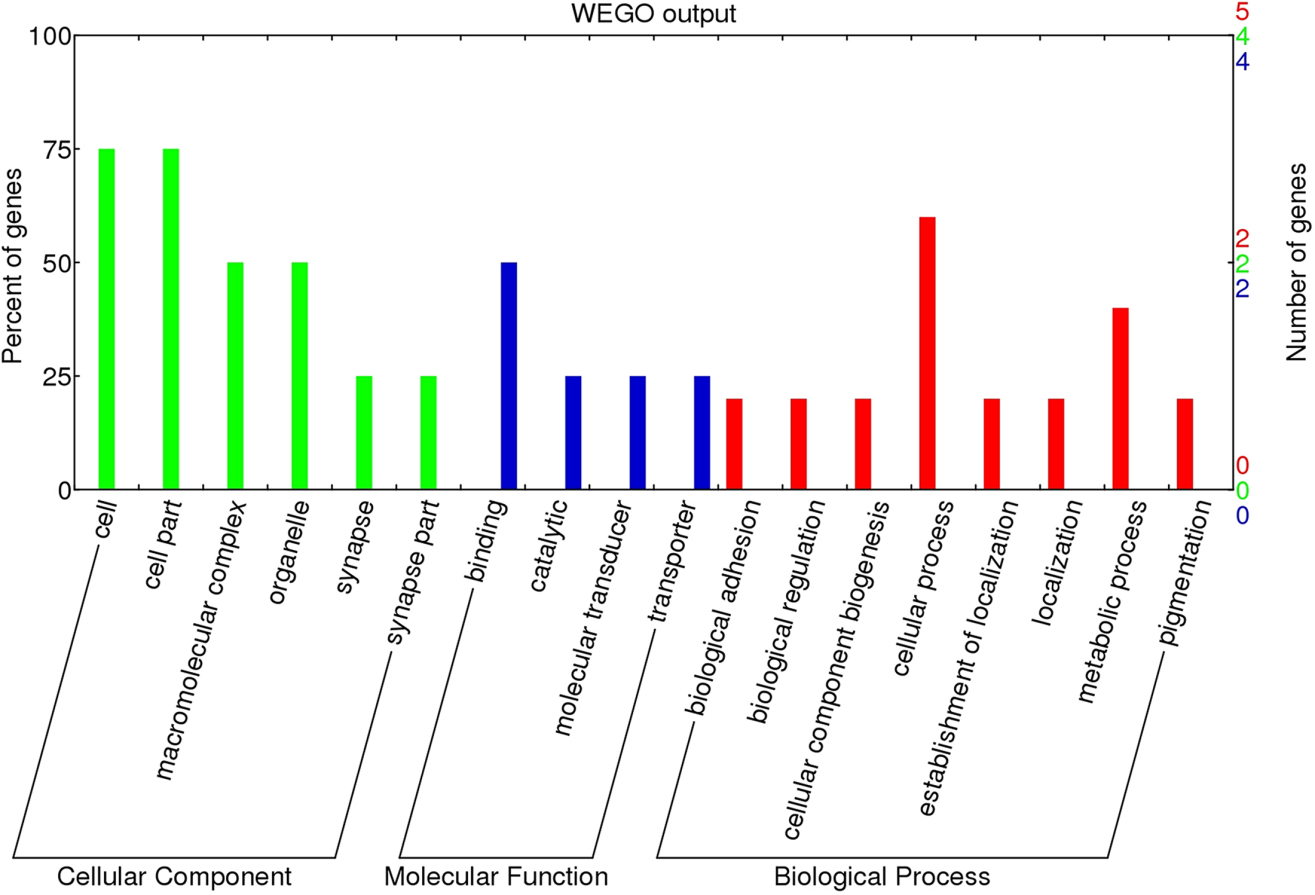
GO analysis of common different expressed piRNAs.

We mapped 32 piRNAs sequences onto the chicken reference genome (*Gallus gallus* v.4) using Bowtie 2.0. After filtering for repeats, we were able to annotate 9 (marked in red in S3 Table). GO analysis of these genes showed that within the Biological Process category, nearly 60% of genes were related to cellular processes (including *RPL7A*, *PUM1*, *CPXM2)*, while 20% of genes were involved in cellular component biogenesis. Nearly 75% of genes are present in the cell part within the Cellular Component category, and 55% of genes were associated with binding, including ATP binding, nucleotide binding, or metal ion binding among the Molecular Function category (Fig 9). In the KEGG analysis, genes were mainly identified in two pathways: the ribosome pathway and the neuroactive ligand-receptor interaction pathway.

### Potential network of 22 piRNAs genes

**Fig 10:**
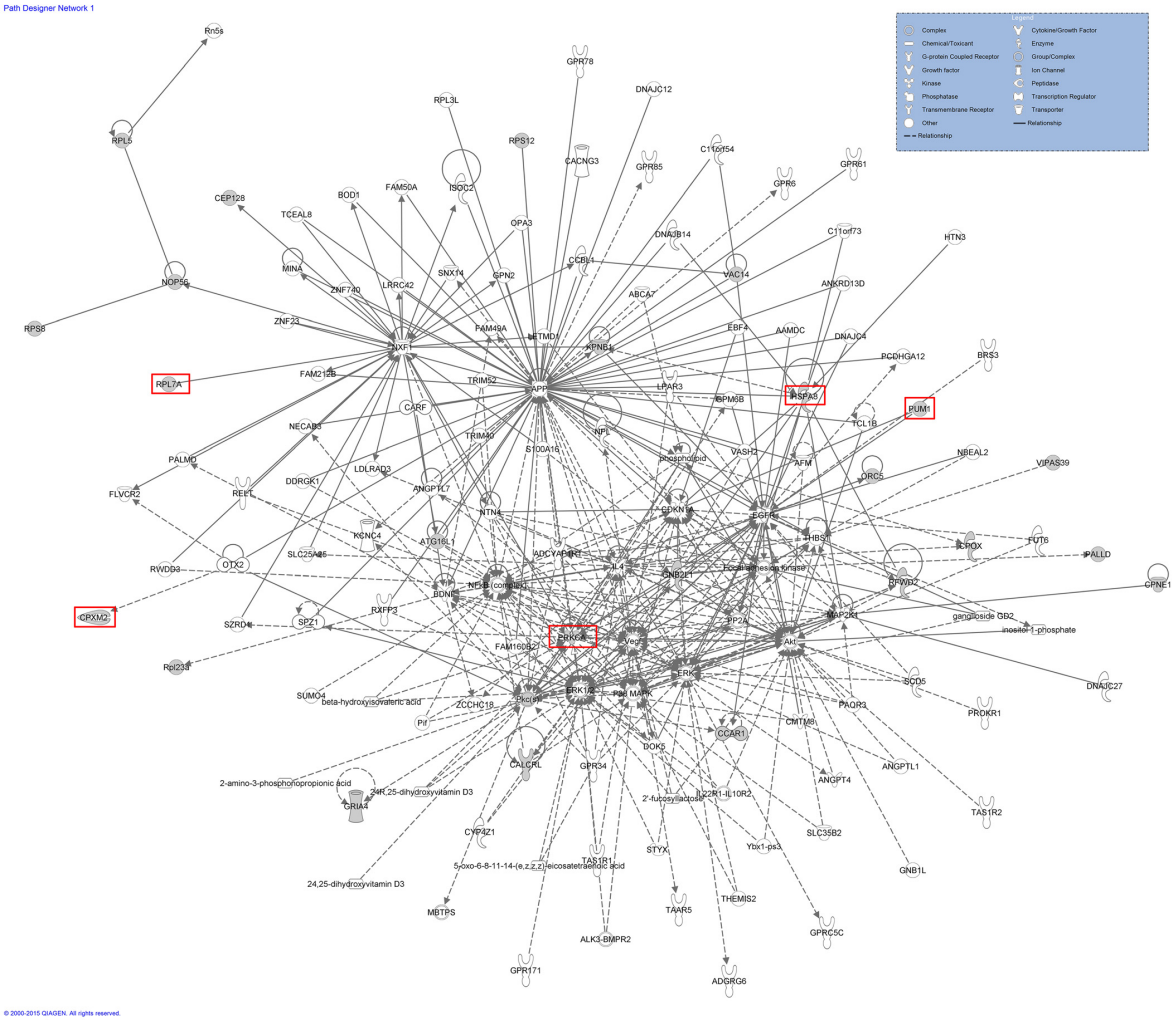
Potential network of 22 piRNAs genes. Different shapes indicate different functions in different pathways. Solid lines and dashed lines indicate direct interactions and indirect interactions, respectively. The red box indicates the 5 genes that were acquired from the GO analysis.

In order to identify a common network among the 22 genes, including the 5 genes, we performed an Ingenuity pathway analysis (IPA) (Fig 10). The results revealed a potential network including the 22 genes. Focusing on the 5 genes we acquired from the GO analysis (*PRKCA*, *EGFR*, *GNB2L1*, *APP*, and *MAPK*), we found that several key elements combined these 5 genes (Fig 10).

### Screening of candidate genes involved in germline stem cell proliferation and spermatogenesis

We searched for candidate genes and pathways with two separate approaches. First, we identified genes with piRNAs common to all three cell types. Using this approach, we identified 22 genes that are involved in processes such as immune system processes, death, pigmentation, and biological regulation. We also acquired 5 genes involved in cellular processes (*RPL7A*, *HSPA8*, *Pum1*, *CPXM2*, and *PRKCA)*. Protein kinase C alpha (*PRKCA*) is a key element with many functions, including the ability to regulate catalytic activity, inactivate MAPK activity, and regulate transferase activity. We speculate that this gene could occupy a central role, involved in inactivating or activating several pathways. Pumilio RNA-binding family member 1 (*Pum1*) is mainly involved in the regulation of cellular protein metabolic processes and translation. We infer this gene performs the same function in germline stem cell proliferation or spermatogenesis. Heat Shock 70kDa Protein 8 (*HSPA8*) is involved in protein folding, and ribosomal Protein L7a (*RPL7A*) mainly participates in ribosome biogenesis according to the GO analysis. Carboxypeptidase X (M14 family) member 2 (*CPXM2*) is related to cell adhesion according to the GO analysis. Using the second approach of identifying the common different piRNAs, results showed that *RPL7A*, *Pum1*, and *CPXM2* were involved in this process. These genes overlap with the genes from the former approach.

According to KEGG analysis, 3 out of 26 pathways were related to embryonic development and stem cell proliferation or spermatogenesis: the Wnt, FGF, and EGF receptor signaling pathways. Furthermore, five pathways were associated with cell differentiation, migration, and programmed cell death, namely the apoptosis, angiogenesis, MAPK, p53, and PDGF pathways.

### RT-qPCR of candidate genes

**Fig 11:**
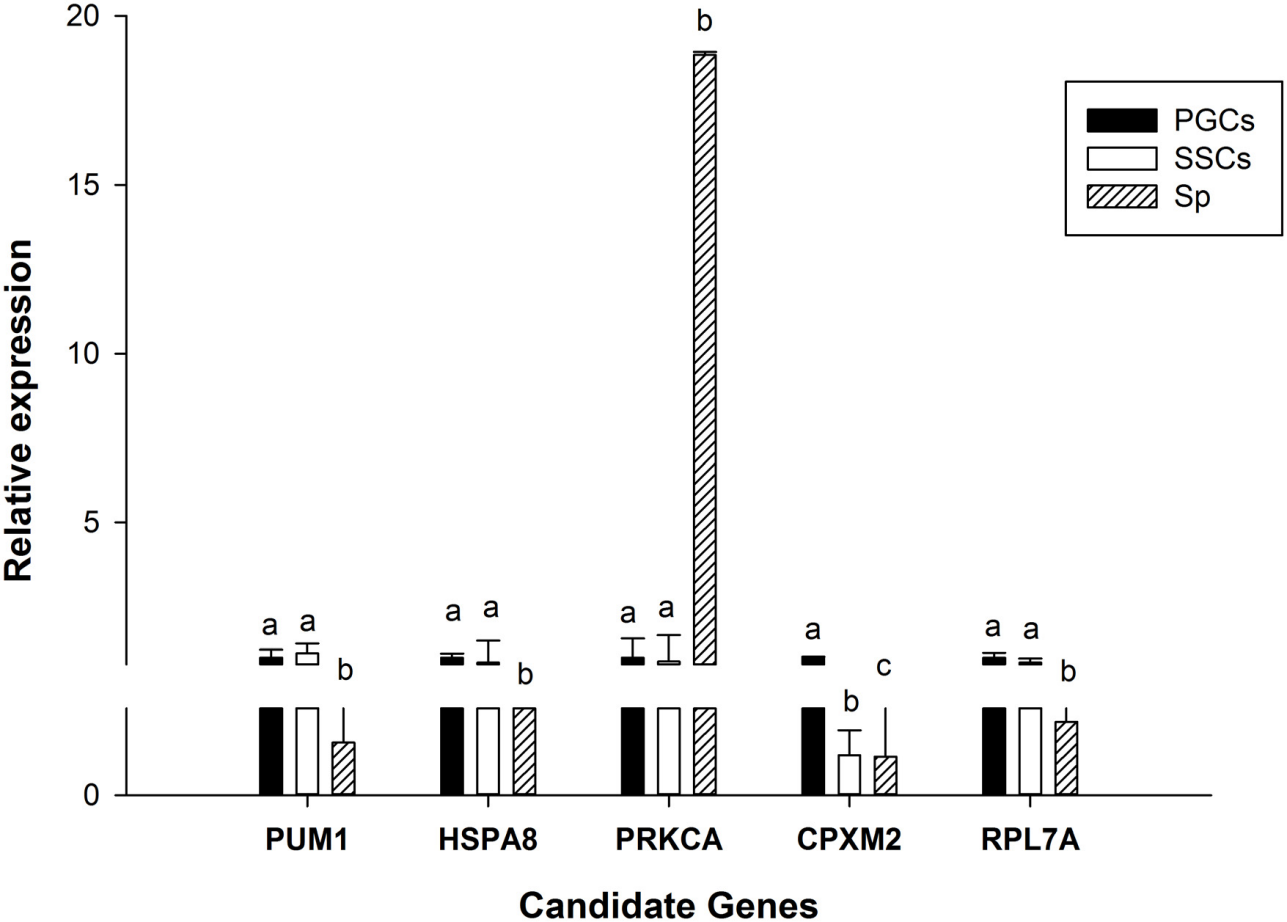
RT-qPCR of candidate genes. Lowercase letters indicated p ≤ 0.01.

We assessed the relative expression of the above 5 candidate genes (Fig 11). The SYBR green primers used are provided in S5 Table. From this analysis, we found that there was no difference between *Pum1*, *HSPA8*, *PRKCA*, and *RPL7A* genes in PGCs and SSCs but in Sp there was significant difference in these genes. The *PRKCA* gene were expressed highest in Sp and lowest in PGCs. Furthermore, except PRKCA, the other three genes expressed higher in PGCs and SSCs than in Sp, indicating that they play a role in the three cell types. On the other hand, *CPXM2* expression showed significant differences in the three cell types. Notably, the expression of *PRKCA* in Sp was much higher than in the other two cell types, indicating that it may act as a key element during spermatogenesis.

## Discussion

Spermatogenesis is a complicated process that requires the coordinated efforts of germ cells and several somatic cells within the tubular structure of the testis [17]. We have examined three cell types in chicken through a small RNA sequencing approach. Through comparison of the results, we revealed that germline stem cell differentiation is a complex regulatory process associated with several genes and signaling pathways.

Germline stem cells are of interest in that they are involved in the passing of genetic information from one generation to the next via sexual reproduction [18]. Following the process of stem cell development and formation of sperm, primordial germ cells (PGCs) and spermatogonial stem cells (SSCs) are the precursors of germ cells and are specified during the early days of embryonic development in birds [19–22]. The first cells involved in spermatogenesis are called spermatogonia, which yield primary spermatocytes via mitosis [23]. GO analysis revealed that the *Pum1* gene is involved in many processes. Several reports have identified that Pum1 plays a role in embryogenesis or spermatogenesis. In haploid ESCs, it was found that Pum1 disruption prompted self-renewal when cells were cultured in conditions allowing for differentiation in mice [24]; however, in mammals, *Pum1* and the p53-mediated pathway act as a guard to maintain germline homeostasis. After removal of *Pum1*, males have a significantly reduced sperm count and reduced fertility, and they display elevated rates of apoptosis in spermatocytes [25]. Furthermore, in mice, the lack of *Pum1* gene trap homozygotes suggested that an early loss of homozygous premplantation embryos because of 96-hr in vitro culture of 1-cell embryos either by natural mating or in vitro fertilization between heterozygotes failed to uncover any homozygous blastocysts [26]. Thus, we infer that *Pum1* plays a similar role in chickens during spermatogenesis. In early spermatogenesis, *Pum1* acts as a marker of non-differentiation in germline stem cells and promotes cell proliferation.

The result of IPA revealed a potential network of 22 genes that is similar to the results of the KEGG analysis implicating the FGF, Wnt, and EGF receptor signaling pathways. Some reports have suggested that these pathways are involved in germline stem cell development. For example, the role of Wnt in embryonic development was discovered when genetic mutations in proteins in the Wnt pathway produced abnormal fruit fly embryos. Later research found that the genes responsible for these abnormalities also influenced breast cancer development in mice [27]. Saito-Diaz et al. found that the Wnt pathway was required for stem cell self-renewal in developing organisms [28]. Therefore, we infer that the Wnt pathway promotes the development and proliferation of germline stem cells (PGCs, SSCs, Sp) in chicken. Fibroblast growth factor (FGF) acts on a variety of different cell types, functioning as both a direct and an indirect stimulator of angiogenesis. It has been reported that members of the FGF family function in the earliest stages of embryonic development and during organogenesis to maintain progenitor cells and mediate their growth, differentiation, survival, and patterning [29]. Choi et al. revealed that basic fibroblast growth factor (bFGF) is one of the key factors that enable proliferation of chicken PGCs via MEK/ERK signaling, regulating downstream genes that may be important for PGC proliferation and survival [30].

piRNAs are a new kind of non-coding RNAs that are important in spermatogenesis. In this study, we found several piRNAs that are associated with early-stage spermatogenesis, and we identified additional piRNAs that could be further studied in the future. In order to further illustrate the role of piRNAs in spermatogenesis, additional research should focus on the next two stages of development: from spermatogonium to primary spermatocyte during the first meiotic division and from primary spermatocyte to secondary spermatocyte during the second meiotic division.

## Conclusions

This study revealed a key gene, *Pum1*, which promotes both spermatogenesis and germline stem cell development. Additionally, we found that the Wnt, FGF, and EGF receptor signaling pathways are important in germ cell development.

## Supporting Information

S1 Table Number of piRNAs classified into 9 groups in three cell types. (DOCX)

S2 Table Sequences of 153 piRNAs. (DOCX)

S3 Table List of 22 genes acquired from 153 sequences. The 9 genes marked in red were acquired by mapping 32 piRNAs sequences.
(XLSX)

S4 Table Sequences of 32 piRNAs. (DOCX)

S5 Table qRT-PCR primers of five candidate genes. (DOCX)
