# Supplementary material for: Discovery of piRNAs Pathway Associated with Early-Stage Spermatogenesis in Chicken
Source: PLoS One. 2016 Apr 5;11(4):e0151780. doi: 10.1371/journal.pone.0151780 (PMC4821617; doi:10.1371/journal.pone.0151780)
Supplement: S1 Table — (DOCX) [file pone.0151780.s001.docx]

Table The number of nine groups classification in three types of cells.

| PGCs | Length(nt) | miRNA | misc_RNA | Mt_rRNA | Mt_tRNA | pseudogene | rRNA | snoRNA | snRNA | Piwi-interacting |
| --- | --- | --- | --- | --- | --- | --- | --- | --- | --- | --- |
|  | 18 | 65091 | 2309 | 1831 | 2775 | 18 | 493322 | 7673 | 6181 | 36859 |
|  | 19 | 64036 | 2046 | 2440 | 3221 | 18 | 243402 | 7410 | 4274 | 76013 |
|  | 20 | 556196 | 2501 | 2912 | 3158 | 12 | 21099 | 5872 | 1165 | 35819 |
|  | 21 | 2827251 | 3138 | 2748 | 2873 | 14 | 17515 | 9065 | 4516 | 37692 |
|  | 22 | 7437471 | 2937 | 2567 | 1868 | 18 | 23197 | 15031 | 2195 | 56207 |
|  | 23 | 3175530 | 2725 | 2721 | 1676 | 22 | 42929 | 29807 | 1018 | 46903 |
|  | 24 | 2956002 | 2358 | 2487 | 2209 | 67 | 96241 | 11209 | 791 | 64933 |
|  | 25 | 295524 | 1578 | 4305 | 1825 | 26 | 33901 | 11896 | 883 | 30014 |
|  | 26 | 22897 | 1580 | 10933 | 1734 | 19 | 33434 | 12802 | 1254 | 49890 |
|  | 27 | 12633 | 2105 | 17159 | 1556 | 23 | 45618 | 11477 | 2386 | 28902 |
|  | 28 | 10855 | 17288 | 16542 | 1868 | 18 | 22618 | 19986 | 2296 | 16458 |
|  | 29 | 12981 | 11549 | 12650 | 6432 | 19 | 20493 | 24951 | 1890 | 13477 |
|  | 30 | 11844 | 4243 | 10244 | 6016 | 10 | 21085 | 26395 | 3026 | 20830 |
|  | 31 | 7943 | 4190 | 7208 | 18646 | 18 | 20092 | 13858 | 2258 | 14563 |
|  | 32 | 6975 | 1515 | 2616 | 9160 | 15 | 21513 | 18584 | 4354 | 32240 |
|  | 33 | 6722 | 1857 | 3884 | 3065 | 27 | 23489 | 13832 | 983 | 21492 |
|  | 34 | 9252 | 1251 | 7476 | 3625 | 16 | 23968 | 21873 | 2914 | 236 |
| SSCs |  |  |  |  |  |  |  |  |  |  |
|  | 18 | 81248 | 2624 | 1608 | 1993 | 24 | 885202 | 19503 | 12668 | 39969 |
|  | 19 | 75803 | 2136 | 1883 | 2564 | 16 | 376985 | 16969 | 8762 | 519707 |
|  | 20 | 601313 | 1556 | 2611 | 2795 | 18 | 35089 | 13029 | 1576 | 40513 |
|  | 21 | 1878531 | 2176 | 2638 | 2405 | 15 | 23048 | 22574 | 10332 | 43442 |
|  | 22 | 7669723 | 2698 | 2072 | 1397 | 13 | 28122 | 37919 | 5278 | 71086 |
|  | 23 | 2873840 | 2437 | 2554 | 1183 | 11 | 44568 | 79420 | 1281 | 55838 |
|  | 24 | 3515174 | 1790 | 2217 | 1729 | 25 | 75280 | 18866 | 832 | 100908 |
|  | 25 | 310810 | 1414 | 4949 | 1415 | 26 | 36355 | 19855 | 1013 | 34704 |
|  | 26 | 24496 | 1183 | 11951 | 1241 | 35 | 31166 | 23807 | 1857 | 52718 |
|  | 27 | 9888 | 1461 | 19751 | 1051 | 12 | 42339 | 17887 | 3556 | 29689 |
|  | 28 | 8981 | 19811 | 16729 | 1347 | 27 | 21570 | 32649 | 2775 | 20642 |
|  | 29 | 8367 | 15822 | 12999 | 2103 | 11 | 18816 | 42412 | 2671 | 17426 |
|  | 30 | 7521 | 3283 | 10120 | 2382 | 16 | 20831 | 39680 | 4446 | 33194 |
|  | 31 | 5078 | 6206 | 6959 | 6407 | 13 | 19295 | 19110 | 4166 | 11183 |
|  | 32 | 4866 | 1373 | 1889 | 3616 | 10 | 23589 | 32278 | 10210 | 30012 |
|  | 33 | 4429 | 1891 | 4017 | 2269 | 9 | 21837 | 25988 | 937 | 14773 |
|  | 34 | 6023 | 1133 | 9094 | 2949 | 19 | 20565 | 46234 | 3080 | 102 |
|  |  |  |  |  |  |  |  |  |  |  |
| Sp | 18 | 1759 | 422 | 908 | 933 | 60 | 174376 | 3959 | 3152 | 22556 |
|  | 19 | 4702 | 707 | 1375 | 1198 | 94 | 174546 | 5900 | 909 | 32779 |
|  | 20 | 31522 | 937 | 1393 | 1555 | 79 | 22555 | 7464 | 1337 | 17140 |
|  | 21 | 131071 | 1308 | 1697 | 1695 | 155 | 27111 | 9386 | 1476 | 31579 |
|  | 22 | 261696 | 1460 | 1686 | 1249 | 246 | 22339 | 19021 | 1522 | 34234 |
|  | 23 | 115056 | 2722 | 2001 | 1489 | 792 | 22514 | 23523 | 1017 | 48190 |
|  | 24 | 77691 | 2892 | 1787 | 2931 | 843 | 66264 | 22274 | 1556 | 44897 |
|  | 25 | 9847 | 3601 | 3141 | 2145 | 1260 | 357963 | 14258 | 2511 | 45364 |
|  | 26 | 6243 | 3237 | 4771 | 2496 | 1163 | 82943 | 14407 | 4124 | 48285 |
|  | 27 | 5578 | 2834 | 15115 | 3784 | 1144 | 52539 | 23630 | 1670 | 22109 |
|  | 28 | 3988 | 2996 | 6861 | 4747 | 644 | 24816 | 26949 | 1509 | 13300 |
|  | 29 | 2587 | 1997 | 7606 | 9124 | 222 | 126372 | 15066 | 1028 | 13773 |
|  | 30 | 2124 | 1518 | 5078 | 12500 | 100 | 36474 | 15081 | 1703 | 14329 |
|  | 31 | 2035 | 1642 | 3183 | 29931 | 101 | 121904 | 16785 | 990 | 79771 |
|  | 32 | 2895 | 1326 | 2677 | 10234 | 65 | 59088 | 14742 | 1304 | 266677 |
|  | 33 | 2215 | 1309 | 4059 | 11872 | 63 | 79640 | 24414 | 837 | 102601 |
|  | 34 | 1785 | 1264 | 5614 | 9249 | 75 | 61846 | 58582 | 598 | 64 |
