# Supplementary material for: Discovery of piRNAs Pathway Associated with Early-Stage Spermatogenesis in Chicken
Source: PLoS One. 2016 Apr 5;11(4):e0151780. doi: 10.1371/journal.pone.0151780 (PMC4821617; doi:10.1371/journal.pone.0151780)
Supplement: S2 Table — (DOCX) [file pone.0151780.s002.docx]

Table 153 piRNA Sequences

| piRNA | sequence |  |
| --- | --- | --- |
| >piRNA_1 | TCCTGGGAATACCGGGTGCTGTAGGCT | |
| >piRNA_10 | TCCCACATCGTCTAGCGGTTAGGATTCCTGGTTT | |
| >piRNA_100 | ACCTCCTGGGAATACCGGGTGCTGTAGGCTT | |
| >piRNA_102 | GCATTGGTGGTTCAGTGGTTGAATTCTCGCCTGC | |
| >piRNA_104 | TCCCATATTGTCTAGCGGTTAGGATTCCTGGT | |
| >piRNA_105 | TTGGTGGTTCAGTGGTAGAATTCTCGCC | |
| >piRNA_106 | TTCTGCCCAGTGCTCTGAATGTCAAAG | |
| >piRNA_107 | TCCCATATGGTCTAGCGGTTAGGATTCCTGG | |
| >piRNA_109 | CCAGGCGAGAATTCTACCACTGAACCACCAATGC | |
| >piRNA_11 | GCATTGGTGGATCAGTGGTAGAATTCTCGCCTGC | |
| >piRNA_110 | TCTGTGGGATTATGACTGAACGCCTCT | |
| >piRNA_111 | GCATTGGTGGTTCAGTGGTAGAATTCTCGCCTCC | |
| >piRNA_113 | GCAGGCAAGAATTCTACCACTGAACCACCAATGC | |
| >piRNA_114 | GCATTGGTGGTTCAGTGGTAGAATTCTCGCA | |
| >piRNA_116 | GCATTGGTGGTTCAGTGGTAGAATTCTCGCCCGC | |
| >piRNA_117 | TCATTGGTGGTTCAGTGGTAGAATTCTCGCCTGC | |
| >piRNA_118 | GAGGCTACCATCTGTGGGATTATGACTGAAC | |
| >piRNA_119 | TCCCATATTGTCTAGCGGTTAGGATTCCTGGTTT | |
| >piRNA_12 | TCTGAAATAGTCTGTGGAAAAAAATAACTGAACT | |
| >piRNA_121 | GCATTGGTGGTTCAGTGGTAGAATTCGCGCCTGC | |
| >piRNA_123 | GCATTTGTGGTTCAGTGGTAGAATTCTCGCC | |
| >piRNA_124 | GCAGGCGAGAATTCTACCACTGAACAACCAATGC | |
| >piRNA_125 | TCTGCTGATCCTCCGGGATCTGAGGACA | |
| >piRNA_126 | GCCTTGGTGGTTCAGTGGTAGAATTCTCGCCT | |
| >piRNA_127 | TTGAGGGATGACTGTTTGGAGATCTGAAG | |
| >piRNA_128 | GCAGGCGAGAATTCCACCACTGAACCACCAATGC | |
| >piRNA_129 | CCTGGGAATACCGGGTGCTGTAGGCTA | |
| >piRNA_130 | TCCCATATTGTCTAGCGGTTAGGATTCCTGGTT | |
| >piRNA_131 | TGCCCAGTGCTCTGAATGTCAAAGTGAAGAA | |
| >piRNA_132 | TGTGATTAGAACTTCTTTGGTAACTGATG | |
| >piRNA_134 | ATCACTCTAGCCAGATCTTGTCTGATG | |
| >piRNA_14 | CAGTCGGATTCCCCTGGTCCGCACCAGT | |
| >piRNA_140 | TAGTACTCTGCGTTGTGGCCGCAGCAACCTCGGT | |
| >piRNA_147 | TTGGTGGTTCAGTGGTAGAATTCTCGCCT | |
| >piRNA_149 | TCCCACATTGTCTAGCGGTTAGGATTCCTGGTT | |
| >piRNA_150 | TCCCATATGGTCTAGTGGTTAGGATTCCTGGT | |
| >piRNA_152 | GAGGCTGATGCCTTATCCATTAGGCAACTGGGTC | |
| >piRNA_153 | GCATTGGTGGTTCAGTTGTAGAATTCTCGCCTGC | |
| >piRNA_154 | GCAGGCGTGAATTCTACCACTGAACCACCAATGC | |
| >piRNA_155 | GCAGGCGAGAATTCTACCACTGAGCCACCAATGC | |
| >piRNA_156 | GCATTGGTGGTTCAGTGGTAGAATTCACGCCTGC | |
| >piRNA_16 | CGGAAACCCAGAGGCTGTTTCTGAGCA | |
| >piRNA_161 | GCAGGCGAGAATTGTACCACTGAACCACCAATGC | |
| >piRNA_162 | GCATTGGTGGGTCAGTGGTAGAATTCTCGCCTGC | |
| >piRNA_163 | GCATTGGTTGTTCAGTGGTAGAATTCTCGCCTGCC | |
| >piRNA_164 | CCTGGGAATACCGGGTGCTGTAGGCTT | |
| >piRNA_165 | GCACTGGTGGTTCAGTGGTAGAATTCTCGCCTGC | |
| >piRNA_167 | TCTGAAATAGTCTGTGGAAAAAAATAACTGAAC | |
| >piRNA_169 | TGAGGGATGACTGTTTGGAGATCTGAAG | |
| >piRNA_171 | GCATTGGTGGTTCAGTGGTGGAATTCTCGCCTGC | |
| >piRNA_174 | GCATTGGTGGTTCAGTGGTAGAATTCTCGCCTGG | |
| >piRNA_175 | TTGGTGGTTCAGTGGTAGAATTCTCGC | |
| >piRNA_176 | AGGCTCAGCTCACAATGAGTCCAGAAGAA | |
| >piRNA_177 | GATCAGCAGTGAGGGCTGTCAGCACAGCG | |
| >piRNA_178 | GCAATGGTGGTTCAGTGGTAGAATTCTCGCCTGC | |
| >piRNA_179 | GCATTGGTTGTTCAGTGGTAGAATTCTCGCCTGC | |
| >piRNA_18 | AAGGCTCAGCTCACAATGAGTCCAGAAGAA | |
| >piRNA_181 | GCAGGCGAGAATTCTACCACTGAACCACAAATGC | |
| >piRNA_182 | GCAAAATGAGCTTTTTAACACTGAGCA | |
| >piRNA_185 | TTGAGGGATGACTGTTTGGAGATCTGAAGA | |
| >piRNA_186 | TCCCACATGGTCTAGCGGTTAGGATTCCTGGT | |
| >piRNA_187 | GCATTGGTGGTTCAGAGGTAGAATTCTCGCCTGC | |
| >piRNA_19 | GGAAACCCAGAGGCTGTTTCTGAGCAC | |
| >piRNA_190 | TTCTGGTGATGAGACCTTTGTCCAGTTCTGCTA | |
| >piRNA_192 | GCATTTGTGGTTCAGTGGTAGAATTCTCGCCTGC | |
| >piRNA_193 | GCATTGGTGGTTCAGTGGTAGAATTCTCGCCAGC | |
| >piRNA_194 | ACCTCCTGGGAATACCGGGTGCTGTAGGCT | |
| >piRNA_196 | GCTGGCGAGAATTCTACCACTGAACCACCAATGC | |
| >piRNA_198 | TCCCATATGGTCTAGTGGTTAGGATTCCTGGTT | |
| >piRNA_199 | GCATTGGTAGTTCAGTGGTAGAATTCTCGCCTGCC | |
| >piRNA_2 | TACCATCTGTGGGATTATGACTGAACGCCT | |
| >piRNA_200 | ATCACTCTAGCCAGGTCTTGTCTGATG | |
| >piRNA_201 | GCATTGGTTGTTCAGTGGTAGAATTCTCGCC | |
| >piRNA_202 | GCATTGGTGGTTCAGTGGCAGAATTCTCGCCTGC | |
| >piRNA_204 | GCAGTGGTGGTTCAGTGGTAGAATTCTCGCCTGC | |
| >piRNA_205 | GGAGACCTCCTGGGAATACCGGGTGCTGTAGGCT | |
| >piRNA_206 | GCAGGCGAGAATTCAACCACTGAACCACCAATGC | |
| >piRNA_207 | GCAGGCGAGAATTCTACCACTGAACCACCAAGGC | |
| >piRNA_208 | TCCCACATTGTCTAGCGGTTAGGATTCCTGGTTT | |
| >piRNA_209 | TTGGTGGTTCAGTGGTAGAATTCTCGCCTGC | |
| >piRNA_21 | TCCCATATGGTCTAGTGGTTAGGATTCCTGGTTT | |
| >piRNA_211 | GCATTGGTGGTTCAGTGGTAGAATTCTCACCTGC | |
| >piRNA_212 | TCCCATATGGTCTAGCGGTTAGGATTCCTGGT | |
| >piRNA_214 | GTCTGTGATGAATAGTAAAAGGTCTGATT | |
| >piRNA_216 | TGCTGTGATGAAACTTCGATGCCATCTGACT | |
| >piRNA_217 | GCAGGCGAGAATTCTACCTCTGAACCACCAATGC | |
| >piRNA_219 | TCCCACATGGTCTAGCGGTTAGGATTCCTGG | |
| >piRNA_220 | GCATTGGTGGTTCAGTGGTAGAATTCTCGCCTTC | |
| >piRNA_221 | GCATTAGTGGTTCAGTGGTAGAATTCTCGCCTGC | |
| >piRNA_222 | GAGGCTACCATCTGTGGGATTATGACT | |
| >piRNA_224 | GAGCGCCAAATCCTAACCACTAGACCACCAGGGA | |
| >piRNA_226 | TAAACAGTCGGATTCCCCTGGTCCGCA | |
| >piRNA_24 | GCGTTGGTGGTTCAGTGGTAGAATTCTCGCCTGC | |
| >piRNA_25 | TTGCAATGATGAAAGTGATTCCTGTGCC | |
| >piRNA_26 | TCCCACATGGTCTAGCGGTTAGGATTCCTGGTT | |
| >piRNA_27 | ATTTCTGCCCAGTGCTCTGAATGTCAAAGT | |
| >piRNA_28 | GCAGGCGAGAATTCTGCCACTGAACCACCAATGC | |
| >piRNA_29 | GCATTGGTGGTTCAGTGGTACAATTCTCGCCTGC | |
| >piRNA_3 | CAGTACTCTGCGTTGTGGCCGCAGCAACCTCGGT | |
| >piRNA_32 | TCTGCCCAGTGCTCTGAATGTCAAAGT | |
| >piRNA_33 | CCCTGTGCGGAAACCCAGAGGCTGTTTCTGAGCA | |
| >piRNA_34 | AGGCTCAGCTCACAATGAGTCCAGAAGAAGC | |
| >piRNA_35 | GCATTTGTGGTTCAGTGGTAGAATTCTCGCCTGCC | |
| >piRNA_36 | GCATTGGTGGTTCAGTGGTAGAATTCTCGCCTGCT | |
| >piRNA_37 | GCATTGGTGGTTCGGTGGTAGAATTCTCGCCTGC | |
| >piRNA_38 | GCATTGGTGGTTCAGTGGTAGAATTCTCGCTTGC | |
| >piRNA_40 | TACCCTCAACAGACAAGGTCGCCTTGACCA | |
| >piRNA_41 | GTCCTGCAATTCACATTAATTCTCGCA | |
| >piRNA_42 | TACCATCTGTGGGATTATGACTGAACGCC | |
| >piRNA_43 | TGCCCAGTGCTCTGAATGTCAAAGTGAAGA | |
| >piRNA_44 | GCTTTGGTGGTTCAGTGGTAGAATTCTCGCCTGC | |
| >piRNA_45 | GCATTAGTGGTTCAGTGGTAGAATTCTCGCCTGCC | |
| >piRNA_46 | GGCAGTGATGTATGAATTTCTTCACCTGAGC | |
| >piRNA_49 | TCCCATATCGTCTAGCGGTTAGGATTCCTGGTTT | |
| >piRNA_5 | TCCCACATGGTCTAGCGGTTAGGATTCCTGGTTT | |
| >piRNA_50 | GAGGCTACCATCTGTGGGATTATGACTGAACGCC | |
| >piRNA_51 | TCCCATATTGTCTAGCGGTTAGGATTCCTGGTTTT | |
| >piRNA_52 | GCATTGGTGGTTCAGCGGTAGAATTCTCGCCTGC | |
| >piRNA_53 | TCCCATATGGTCTAGCGGTTAGGATTCCTGGTT | |
| >piRNA_54 | GCATTAGTGGTTCAGTGGTAGAATTCTCGCC | |
| >piRNA_55 | GCAGGCGAGAATTCTACCACTGAACCACCACTGC | |
| >piRNA_57 | TATCAGAGCCAAACCCTCATCACAGCC | |
| >piRNA_58 | GCATTGGTGGTTCTGTGGTAGAATTCTCGCCTGC | |
| >piRNA_60 | GCATTGGTGGTTCAGTGGTAGAATTCTCGCCTAC | |
| >piRNA_61 | GCAGGCGAGAATTCTACCACTGAACCACCATTGC | |
| >piRNA_63 | GCAGGCGAGAATTCTACCACTGAACTACCAATGC | |
| >piRNA_64 | AAGGCTCAGCTCACAATGAGTCCAGAAGAAGCTC | |
| >piRNA_66 | GCATTGGTAGTTCAGTGGTAGAATTCTCGCCTGC | |
| >piRNA_67 | TCCCATATGGTCTAGCGGTTAGGATTCCTGGTTT | |
| >piRNA_68 | GCAGGCGAGAATTCTACCACTGAACCACCAGTGC | |
| >piRNA_70 | CTCACTGAACAGAGATGAAAACCTAAGGTCTGAGT | |
| >piRNA_72 | GATCAGCAGTGAGGGCTGTCAGCACAGCGGCTCAG | |
| >piRNA_73 | GCATTGGTGGTTCAGTGGTATAATTCTCGCCTGC | |
| >piRNA_74 | TCCCTGTGCGGAAACCCAGAGGCTGTTTCTGAGC | |
| >piRNA_75 | GAGGCTGATGCCTTATCCATTAGGCCACTGGGTC | |
| >piRNA_76 | TTGCAATGATGAAAGTGATTCCTGTGC | |
| >piRNA_79 | GCCTTGGTGGTTCAGTGGTAGAATTCTCGCCTGC | |
| >piRNA_8 | TTTGAGGGATGACTGTTTGGAGATCTGAAG | |
| >piRNA_80 | TTCTGCCCAGTGCTCTGAATGTCAAAGT | |
| >piRNA_82 | CTGCTGTGATGAAACTTCGATGCCATCTGACTGC | |
| >piRNA_85 | GCATTGGTGGCTCAGTGGTAGAATTCTCGCCTGC | |
| >piRNA_87 | GCATTGGTAGTTCAGTGGTAGAATTCTCGCC | |
| >piRNA_88 | TCTGCCCAGTGCTCTGAATGTCAAAGTGAAGA | |
| >piRNA_89 | CTGCTGTGATGAAACTTCGATGCCATCTGACTG | |
| >piRNA_9 | GCATTGGTGGTTTAGTGGTAGAATTCTCGCCTGC | |
| >piRNA_90 | GCAGGTGAGAATTCTACCACTGAACCACCAATGC | |
| >piRNA_92 | GCATTGGTGGTTCAGTGGTAGAATTCTTGCCTGC | |
| >piRNA_93 | GAGACCTCCTGGGAATACCGGGTGCTGTAGGCTT | |
| >piRNA_94 | CTGCTGTGATGAAACTTCGATGCCATCTGACT | |
| >piRNA_96 | GCATTGGTGGTTCAGGGGTAGAATTCTCGCCTGC | |
| >piRNA_97 | TCCCTGTGCGGAAACCCAGAGGCTGTTTCTGAGCA | |
| >piRNA_98 | CTGAACAGAGATGAAAACCTAAGGTCTGAGT | |
| >piRNA_99 | CTGGAAGCTGTTGGGACCAGTTGGGACCGGT | |
