# Supplementary material for: Discovery of piRNAs Pathway Associated with Early-Stage Spermatogenesis in Chicken
Source: PLoS One. 2016 Apr 5;11(4):e0151780. doi: 10.1371/journal.pone.0151780 (PMC4821617; doi:10.1371/journal.pone.0151780)
Supplement: S4 Table — (DOCX) [file pone.0151780.s004.docx]

Table 32 piRNAs sequences

| gene_id | description | PGCs VS SSCs | | | SSCs VS Sp | | |
| --- | --- | --- | --- | --- | --- | --- | --- |
|  |  | PGCs_Tpm | SSCs_Tpm | Fold_Change | SSCs_Tpm | Sp_Tpm | Fold Change |
| piRNA_101 | TGCGAGAATTCTACCACTGAACCACCAATGC | 857.98 | 201.35 | 4.26 | 201.35 | 60.68 | 3.32 |
| piRNA_114 | GCATTGGTGGTTCAGTGGTAGAATTCTCGCA | 857.98 | 201.35 | 4.26 | 201.35 | 1728.88 | 0.12 |
| piRNA_3 | CAGTACTCTGCGTTGTGGCCGCAGCAACCTCGGT | 1069.69 | 286.36 | 3.74 | 286.36 | 4.41 | 64.89 |
| piRNA_145 | ACCGAGGTTGCTGCGGCCACAACGCAGAGTACTG | 1069.69 | 286.36 | 3.74 | 286.36 | 0.28 | 1038.19 |
| piRNA_195 | AATCAGAATCCCTCAAATCATCATGCCCA | 705.70 | 210.30 | 3.36 | 210.30 | 3.59 | 58.65 |
| piRNA_20 | ACCGAGGTTGCTGCGGCCACAACGCAGAGTACTA | 5716.13 | 2058.21 | 2.78 | 2058.21 | 0.83 | 2487.33 |
| piRNA_140 | TAGTACTCTGCGTTGTGGCCGCAGCAACCTCGGT | 5716.13 | 2058.21 | 2.78 | 2058.21 | 12.41 | 165.82 |
| piRNA_110 | TCTGTGGGATTATGACTGAACGCCTCT | 1429.96 | 684.58 | 2.09 | 684.58 | 10.76 | 63.64 |
| piRNA_46 | GGCAGTGATGTATGAATTTCTTCACCTGAGC | 215.42 | 434.01 | 0.50 | 434.01 | 2.21 | 196.69 |
| piRNA_59 | GCCGAATCCTAACCACTAGACCACCAGGGA | 2447.65 | 5006.82 | 0.49 | 5006.82 | 324.65 | 15.42 |
| piRNA_40 | TACCCTCAACAGACAAGGTCGCCTTGACCA | 133.71 | 286.36 | 0.47 | 286.36 | 577.58 | 0.50 |
| piRNA_190 | TTCTGGTGATGAGACCTTTGTCCAGTTCTGCTA | 200.57 | 447.44 | 0.45 | 447.44 | 39.44 | 11.34 |
| piRNA_129 | CCTGGGAATACCGGGTGCTGTAGGCTA | 137.42 | 308.73 | 0.45 | 308.73 | 33.65 | 9.17 |
| piRNA_47 | GGTCTCAGCTTCTATCAGTCATGCATCACGGAG | 419.70 | 948.57 | 0.44 | 948.57 | 160.53 | 5.91 |
| piRNA_34 | AGGCTCAGCTCACAATGAGTCCAGAAGAAGC | 133.71 | 313.21 | 0.43 | 313.21 | 1.10 | 283.88 |
| piRNA_185 | TTGAGGGATGACTGTTTGGAGATCTGAAGA | 256.28 | 617.46 | 0.42 | 617.46 | 13.24 | 46.64 |
| piRNA_32 | TCTGCCCAGTGCTCTGAATGTCAAAGT | 1407.68 | 3534.75 | 0.40 | 3534.75 | 305.06 | 11.59 |
| piRNA_146 | GCGAGAATTCTACCACTGAACCACCAA | 237.71 | 617.46 | 0.38 | 617.46 | 0.83 | 746.20 |
| piRNA_175 | TTGGTGGTTCAGTGGTAGAATTCTCGC | 237.71 | 617.46 | 0.38 | 617.46 | 0.55 | 1119.30 |
| piRNA_41 | GTCCTGCAATTCACATTAATTCTCGCA | 337.99 | 1029.11 | 0.33 | 1029.11 | 159.98 | 6.43 |
| piRNA_197 | CGCCGAATCCTAACCACTAGACCACCAGGGA | 1077.11 | 3333.41 | 0.32 | 3333.41 | 458.15 | 7.28 |
| piRNA_12 | TCTGAAATAGTCTGTGGAAAAAAATAACTGAACT | 2213.65 | 6921.86 | 0.32 | 6921.86 | 49.10 | 140.98 |
| piRNA_19 | GGAAACCCAGAGGCTGTTTCTGAGCAC | 282.28 | 984.36 | 0.29 | 984.36 | 13.24 | 74.35 |
| piRNA_167 | TCTGAAATAGTCTGTGGAAAAAAATAACTGAAC | 5207.29 | 19204.01 | 0.27 | 19204.01 | 32.00 | 600.20 |
| piRNA_16 | CGGAAACCCAGAGGCTGTTTCTGAGCA | 126.28 | 492.18 | 0.26 | 492.18 | 2.76 | 178.44 |
| piRNA_92 | GCATTGGTGGTTCAGTGGTAGAATTCTTGCCTGC | 304.56 | 1610.77 | 0.19 | 1610.77 | 206.59 | 7.80 |
| piRNA_113 | GCAGGCAAGAATTCTACCACTGAACCACCAATGC | 304.56 | 1610.77 | 0.19 | 1610.77 | 418.15 | 3.85 |
| piRNA_182 | GCAAAATGAGCTTTTTAACACTGAGCA | 152.28 | 805.39 | 0.19 | 805.39 | 2.21 | 364.99 |
| piRNA_169 | TGAGGGATGACTGTTTGGAGATCTGAAG | 178.28 | 1064.90 | 0.17 | 1064.90 | 3.59 | 296.98 |
| piRNA_70 | CTCACTGAACAGAGATGAAAACCTAAGGTCTGAGT | 408.56 | 2541.44 | 0.16 | 2541.44 | 43.86 | 57.95 |
| piRNA_38 | GCATTGGTGGTTCAGTGGTAGAATTCTCGCTTGC | 211.71 | 9481.20 | 0.02 | 9481.20 | 1780.46 | 5.33 |
| piRNA_84 | GCAAGCGAGAATTCTACCACTGAACCACCAATGC | 211.71 | 9481.20 | 0.02 | 9481.20 | 395.26 | 23.99 |
