# Supplementary material for: Discovery of piRNAs Pathway Associated with Early-Stage Spermatogenesis in Chicken
Source: PLoS One. 2016 Apr 5;11(4):e0151780. doi: 10.1371/journal.pone.0151780 (PMC4821617; doi:10.1371/journal.pone.0151780)
Supplement: S5 Table — (DOCX) [file pone.0151780.s005.docx]

Table The primer of 5 candidate genes

| No. | Gene Name | Primer |
| --- | --- | --- |
| 1 | *PUM1* | Forward: ACGAGATGGTAC-GGGAGTTG  Reverse: AGTGTGCTGGTGAAGCTCCT |
| 2 | *PRKCA* | Forward: TTCCAAACAGTTGACCGCCT  Reverse: AACACTGCTTGTGGCTCCTT |
| 3 | *RPL7A* | Forward: CACACAAGTTAACCCGGAGGAT  Reverse: CAAGACATTACCGCCCCAGT |
| 4 | *HSPA8* | Forward: GCCGCTCAGACAAGATGTCA  Reverse: CTTGGCGTGGTCCTGTTACC |
| 5 | *CPXM2* | Forward: GAGGAGTGGGAGAACAACCG  Reverse: TCAGCTCCTGTGCGAATGTC |
